# Effects of acupuncture on postoperative recovery and extubation time: A protocol for systematic review and meta analysis

**DOI:** 10.1097/MD.0000000000024502

**Published:** 2021-01-29

**Authors:** Seungwon Kwon, Chul Jin, Aram Jeong, Seung-Bo Yang

**Affiliations:** a Department of Cardiology and Neurology, Kyung Hee University, Seoul; b Department of Pediatrics; c Department of Korean Internal Medicine, College of Korean Medicine, Gachon University, Seongnam-si, Republic of Korea.

**Keywords:** acupuncture, extubation, postoperative, surgery

## Abstract

**Background:**

This systematic review protocol aims to provide evidence of the efficacy and safety of acupuncture on postoperative recovery and extubation time.

**Methods:**

The following 11 electronic databases will be searched from inception: The Cochrane Database of Systematic Reviews, MEDLINE, EMBASE, AMED, CINAHL, 1 Chinese database (CNKI), and 5 Korean databases (OASIS, DBpia, RISS, KISS, NDSL). Only randomized controlled trials of acupuncture treatment for postoperative recovery after surgery will be included for review. The selection of the studies, data extraction, and management will be performed independently by 3 researchers. Methodological quality, including the risk of bias, will be assessed using the Cochrane risk of bias assessment tool.

**Results and Conclusions:**

Our systematic review will provide evidence of the efficacy of acupuncture on postoperative recovery and extubation time. This evidence will provide useful information to practitioners and patients in the field of surgery and complementary medicine.

**PROSPERO registration number:**

2020 CRD42020168411

## 1 Introduction

### 1.1 Description of the conditions

Surgery, also called “surgical procedure” or “operation”, is defined as operative manual and instrumental techniques on a person to treat a pathological condition. After surgery, it is important to awake from anesthesia normally and restore physical function. In addition, it is important to manage the various symptoms that could occur after surgery. Surgical injury can be followed by pain, nausea, vomiting and ileus, stress-induced catabolism, impaired pulmonary function, increased cardiac demands, and risk of thromboembolism. These problems can lead to complications, need for treatment in the hospital, postoperative fatigue, and delayed convalescence.^[1]^

The indicators of postoperative recovery include Bispectral Index, time to spontaneous eye opening, time to tracheal extubation, time from extubation to “ready for discharge” from the Post Anesthesia Care Unit, and time to following commands.^[2,3]^ In addition, laboratory markers such as inflammation levels^[4]^ and brain tissue damage markers,^[5]^ are also used as postoperative recovery indicators.

### 1.2 Description of the intervention

Acupuncture has been practiced for more than 3000 years to treat various symptoms or diseases and is generally the most widely used technique to control pain. Acupuncture is also used to treat various types of diseases such as mental disorders, immune system dysfunctions, and gastrointestinal, gynecological, and neurological diseases.^[6]^

There are various types of acupuncture treatments including dry needling, electroacupuncture, and intradermal acupuncture. For patients undergoing surgery, acupuncture treatment could be used in all periods of surgery, such as before,^[7]^ during,^[8]^ and after surgery.^[3]^

Acupuncture treatment could reduce the amount of anesthesia needed during surgery, thereby reducing adverse effects caused by anesthesia. A recent meta-analysis showed that the use of acupuncture reduced the amount of volatile anesthetics administered during craniotomy and postoperative patient recovery.^[5]^ Acupuncture could also improve postoperative pain and reduce opioid use^[9]^ and might be beneficial in the prevention and treatment of postoperative nausea and vomiting.^[10]^ Furthermore, acupuncture might alleviate excessive inflammation after surgery by reducing levels of interleukin (IL)-1 β,^[11]^ IL-6,^[12]^ and S100β.^[5]^ Therefore, acupuncture might be helpful to relieve postoperative symptoms such as delirium, pain, nausea, and vomiting and enhance postoperative recovery.

Currently, acupuncture treatments have been widely used for postoperative recovery. However, there is no critically appraised evidence of the potential benefit of acupuncture for postoperative recovery. Comprehensive evaluation of postoperative recovery, including the effectiveness of treatment for various symptoms that might appear after surgery, will help in postoperative patient management.

### 1.3 Objectives

This study aims to evaluate the efficacy and safety of acupuncture on postoperative recovery and extubation time.

## 2 Methods

### 2.1 Study registration

The protocol of this systematic review was registered on PROSPERO 2020 (registration number: CRD42020168411).

### 2.2 Inclusion criteria for study selection

#### 2.2.1 Type of studies

Only randomized controlled trials of acupuncture treatment for postoperative recovery after surgery will be included for review. Non-randomized clinical studies, observational studies, case studies, qualitative studies, and laboratory studies will be excluded. Trials that fail to provide detailed results will also be excluded. There are no restrictions depending on the language of writing.

#### 2.2.2 Type of participants

Patients who underwent surgery regardless of age, sex, ethnicity, type of anesthesia, or types of surgery.

#### 2.2.3 Types of interventions and controls

We will include acupuncture treatments performed using various techniques. We will include all types of acupuncture treatments performed before, during, and after surgery, with no limit on the choice of acupoint and methods of acupoint stimulation. Traditional acupuncture, warm needling, and electroacupuncture will be included among methods of acupuncture point stimulation.

Interventions in the control or comparison groups will include no intervention (routine care only), or placebo combined with routine care. Routine care refers to the management usually performed after surgery.

#### 2.2.4 Type of outcome measures

Primary outcomes:

1. total time from the end of surgery to extubation

Secondary outcomes:

1. The amounts of volatile anesthetics (mg) administered and the depth of sedation, such as bispectral index, during the intraoperative period
2. Reaction times (minutes) during emergence
3. Postoperative analgesic consumption and pain intensity (VAS) during the postoperative period
4. Inflammatory and immune laboratory levels (e. g., IL-1β, IL-6, tumor necrosis factor -α, and S100β protein)
5. Quality of life (e.g., The Short Form 36 Health Survey, and EuroQol 5 dimension scale
6. Number and severity of adverse events.

### 2.3 Data sources

The following 11 electronic databases will be searched from inception to December 2019: The Cochrane Database of Systematic Reviews, MEDLINE, Excerpta Medica dataBASE (EMBASE), Allied and Complementary Medicine Database (AMED), Cumulative Index to Nursing and Allied Health Literature (CINAHL), 1 Chinese database (China Academic Journals full-text database [CNKI]), and 5 Korean databases (Online Acquisitions and Selection Information System [OASIS], DBpia, Research Information Service System [RISS], Korean Information Service System [KISS], National Discovery for Science Leaders [NDSL]). Bibliographic references will be investigated manually to avoid missing eligible trials.

### 2.4 Search strategy

Our search strategy will include the main keywords “acupuncture” and “surgery”. The search strategy for MEDLINE is shown in Table 1. The search words used in the Chinese and Korean databases have the same meaning as those used in the English databases.

**Table 1:** Search strategy used in MEDLINE database.

Number	Search terms
1	“Clinical Trials as Topic” [MeSH]
2	randomized controlled trial [pt]
3	controlled clinical trial [pt]
4	randomized controlled trial [MeSH]
5	randomized allocation [MeSH]
6	randomized [tiab]
7	randomised [tiab]
8	placebo [tiab]
9	randomly [tiab]
10	trial [tiab]
11	or 1–10
12	animals [MeSH] NOT humans [MeSH]
13	11 NOT 12
14	surgery [tiab]
15	“surger^∗^.” [tiab]
16	“surgion^∗^.” [tiab]
17	surgical [tiab]
18	operation [tiab]
19	operative [tiab]
20	postoperative [tiab]
21	perioperative [tiab]
22	exp Surgical Procedures, Operative/
23	exp Specialties, Surgical/
28	or 14–23
29	acupuncture [MeSH]
30	acupuncture therapy [MeSH]
31	acupuncture [tiab]
32	acupuncture therapy [tiab]
33	manual acupuncture [tiab]
34	electroacupuncture [tiab]
35	electro-acupuncture [tiab]
36	electroacupuncture therapy [tiab]
37	dry needle [tiab]
38	fire needling [tiab]
39	fire needle [tiab]
40	fire acupuncture [tiab]
41	warm needling [tiab]
42	warming needle moxibustion [tiab]
43	moxibustion acupuncture [tiab]
44	pyonex [tiab]
45	body acupuncture [tiab]
46	scalp acupuncture [tiab]
47	auricular acupuncture [tiab]
48	ear acupuncture [tiab]
49	intradermal needling [tiab]
50	or 29–49
51	13 and 28 and 50

This search strategy will be modified as required for other electronic databases.

### 2.5 Data collection and analysis

#### 2.5.1 Study selection

**Figure 1:**
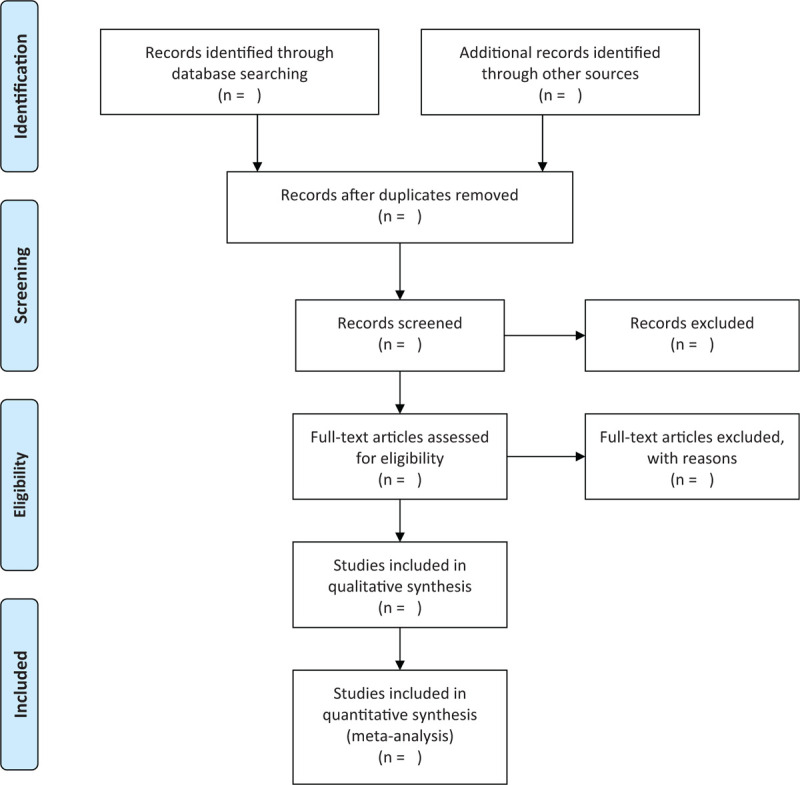
Study selection flow diagram.

Three authors (SK, CJ, and AJ) will independently screen the titles and abstracts of eligible studies. Disagreements will be resolved through discussions between all authors. When disagreements on the selection cannot be resolved through discussions, the arbiter (SBY) will make a final decision. Details on study selection will be presented in a Preferred Reporting Items for Systematic Reviews and Meta-Analyses (PRISMA) flow diagram (Fig. 1).

#### 2.5.2 Data extraction and management

Data extraction and quality assessment will be conducted independently by 3 authors (SK, CJ, AJ, and SBY). The extracted data will be written in a standard form (e.g., authors, study design, participants, control, intervention, outcome measures, and results). If there is a disagreement regarding the extracted data between the authors, a consensus will be reached at a meeting between all authors. If there are insufficient data, SBY will contact the original study author via email or telephone to request additional information.

#### 2.5.3 Assessment of risk of bias in the included studies

We will independently assess the risk of bias to evaluate the methodological quality of the included studies, according to the criteria from the Cochrane Handbook.^[13]^ The following domains will be assessed: sequence generation, allocation concealment, blinding of participants and outcome assessors, incomplete outcome data, and selective outcome reporting. The risks of bias will be categorized into 3 levels: “Low,” “High”, or “Unclear” according to Cochrane guideline criteria. If there is any difference in opinion between the authors, a consensus will be reached at a meeting between all authors.

#### 2.5.4 Measures of the treatment effect

For continuous data, the mean difference will be used with 95% confidence intervals (CIs) to measure the treatment effect. If methods or scales are not the same, we will use the standardized mean difference with 95% CIs. For dichotomous data, the risk ratio will be used with 95% CIs to measure the treatment effect.

#### 2.5.5 Unit of analysis issues

The data at the time of extubation will be analyzed. In addition, the data at the time of discharge from the Intensive Care Unit and hospital will also be analyzed. If there are multiple time point observations, the data will be analyzed as either a short-term (within 1 week) or long-term (over 1 week) follow-up.

#### 2.5.6 Dealing with missing data

In studies with missing data, we will contact the author of the original article to request these data. If we are unable to contact the author, we will analyze only the available data.

#### 2.5.7 Assessment of heterogeneity

Fixed-effects and random-effects models will be used for the meta-analysis according to the data analysis. Heterogeneity will be tested with the I^2^ test, using 50% as the cutoff point for meaningful heterogeneity. If heterogeneity is observed, we will conduct a subgroup analysis to explore the possible causes.^[13]^

#### 2.5.8 Assessment of reporting bias

When more than 10 studies are available, funnel plots will be used to detect reporting biases and Egger regression test will be used to determine funnel plot asymmetry.^[13,14]^

#### 2.5.9 Data synthesis

We will perform the meta-analysis using Review Manager software (RevMan, V.5.4 for Windows; the Nordic Cochrane Centre, Copenhagen, Denmark) to assess the differences between the intervention and control groups. The risk ratio and 95% CIs will be assessed for the effect size of each included study. The fixed-effects model will be used for pooled data if no substantial statistical heterogeneity is detected. The random-effects model will be used if there is substantial statistical heterogeneity. The studies will be synthesized according to the type of intervention and/or control as follows:

i) acupuncture + postoperative routine care vs. no treatment + postoperative routine care
ii) acupuncture + postoperative routine care vs. placebo control + postoperative routine care

#### 2.5.10 Subgroup analysis and investigation of heterogeneity

When sufficient numbers of studies are available, subgroup analyses will be conducted on the following topics:

1. Type of acupuncture (e.g., manual acupuncture, electroacupuncture, or ear acupuncture)
2. Timing of acupuncture treatment (e.g., before, during, or after surgery or a combination thereof)
3. Type of control (e.g., no treatment, sham acupuncture)
4. 4. Type of surgery

#### 2.5.11 Sensitivity analysis

When sufficient numbers of studies are available, sensitivity analysis will be conducted according to the following criteria:

1. Methodological qualities (sequence generation, allocation concealment, or blinding)
2. Sample size (e.g., less than 40 participants in each group)

#### 2.5.12 Quality of evidence

The quality of evidence for all outcomes will be assessed through the Grading of Recommendations Assessment Development and Evaluation approach. We will categorize the quality of evidence into 4 levels: high, moderate, low, and very low quality.

#### 2.5.13 Ethical approval

Ethical approval is not necessary because this study will be based on published research.

## 3 Discussion

This systematic review will provide evidence of the efficacy of acupuncture on postoperative recovery and extubation time. Various indicators could be used to evaluate postoperative recovery. In this study, the total time from the end of surgery to extubation will be analyzed as the primary outcome. Various outcome measures will also be analyzed, including the amount of volatile anesthetics administered and the depth of sedation during the intraoperative period, reaction times during emergence, postoperative analgesic consumption and pain intensity during the postoperative period, and inflammatory and immune marker levels.

This analysis will include patients who underwent surgery, regardless of patient age, sex, or ethnicity as well as the type of anesthesia or surgery. We will include all types of acupuncture treatments performed before, during, and after surgery.

This evidence will provide useful information to practitioners and patients in the field of surgery and complementary medicine.

## Author contributions

The search strategy was developed by SBY. SK, CJ, and AJ will search and select the studies. SBY will act as an arbiter in the selection stage. Extraction of data, assessment of risk of bias, and reporting quality and quality of evidence will be performed by all authors (SK, CJ, AJ, SBY). Interpretation of the analyses will be performed by all authors (SK, CJ, AJ, SBY). All authors read and approved the final manuscript for publication (SK, CJ, AJ, SBY).

**Conceptualization:** Seung-Bo Yang.

**Data curation:** Seungwon Kwon, Chul Jin, Aram Jeong.

**Funding acquisition:** Seung-Bo Yang.

**Investigation:** Seungwon Kwon, Chul Jin.

**Methodology:** Seungwon Kwon, Aram Jeong, Seung-Bo Yang.

**Supervision:** Seung-Bo Yang.

**Validation:** Seung-Bo Yang.

**Writing – original draft:** Seungwon Kwon, Seung-Bo Yang.

**Writing – review & editing:** Seung-Bo Yang.

## Abbreviations

Abbreviations: IL = interleukin, TNF = tumor necrosis factor.
